# Complete genomes of the hydrogenotrophic methanogen species*—Methanospirillum stamsii* Pt1 and *Methanospirillum lacunae* Ki8-1

**DOI:** 10.1128/mra.00736-25

**Published:** 2025-08-27

**Authors:** Ethan Humm, Rajshree Chettiyar, Liudmilla Rubbi, Giorgia Del Vecchio, Matteo Pellegrini, Sung Min Ha, Robert P. Gunsalus

**Affiliations:** 1 Department of Microbiology, Immunology and Molecular Genetics, University of California 8783, Los Angeles, California, USA; 2 Department of Molecular, Cell and Developmental Biology, University of California 8783, Los Angeles, California, USA; 3 UCLA DOE Institute, University of California 8783, Los Angeles, California, USA; 4 Department of Integrative Biology and Physiology, University of California 8783, Los Angeles, California, USA; California State University San Marcos, San Marcos, California, USA

**Keywords:** *Methanospirillum*, genomes, methanogens, syntrophs

## Abstract

*Methanospirillum* species grow within sheaths containing 1–50 or more cells and often in association with syntrophic bacteria that together metabolize a variety of aliphatic and aromatic compounds. Here, we report the complete genomes of *Methanospirillum stamsii* Pt1 *and Methanospirillum lacunae* Ki8-1.

## ANNOUNCEMENT

The *Methanospirillaceae* are obligate hydrogenotrophic methanogens (1) that inhabit a variety of anaerobic habitats worldwide. *Methanospirillum lacunae* Ki8-1 is a mesophilic strain isolated from freshwater puddle soil, whereas the taxonomically related strain, *Methanospirillum stamsii* Pt1, is psychrotolerant and isolated from a propionate-oxidizing methanogenic consortium (2, 3). Both are spirilla-shaped and grow within a proteinaceous sheath with lengths up to 0.5 µm or more. Their genomes provide insight into the ability to perform symbiotic co-operations with a variety of syntrophic bacteria (4, 5) and inform about the habitats and relationships of methanogen species.

*M. lacunae* Ki8-1 (DSM 22751) and *M. stamsii* Pt1 (DSM 26304) were obtained from the DSMZ culture collection and grown statically at 30℃ in 125 mL serum bottles containing anaerobic mineral salts media and a headspace pressurized with an 80:20 (vol/vol) H_2_:CO_2_ mixture. *M. lacunae* Ki8-1 was cultured in DSMZ medium 1273 (https://www.dsmz.de/microorganisms/medium/pdf/DSMZ_Medium1273.pdf). *M. stamsii* Pt1 was cultured in the medium described by Wang et al. (6), with the following modifications: addition of 1 g/L yeast extract and 2 g/L sodium formate, and 10-fold reduction of NaCl, K_2_HPO_4_, KH_2_PO_4_, and NH_4_Cl. Cells were grown to mid-log phase, harvested, and the DNA was extracted using the Zymo D6060 Quick-DNA HMW Magbead Kit as per the manufacturer’s instructions and fragmented using Covaris g-TUBEs following instructions from the manufacturer. The average size of the sheared gDNA was checked at the TapeStation 4200 (Agilent). Multiplexed microbial libraries were prepared using the PacBio SMRTbell Prep Kit 3.0 together with the SMRTbell barcoded adapters 3.0 according to the PacBio protocol. Final whole genome libraries were not size-selected but simply purified via a standard procedure using 1X SMRTbell clean-up beads. DNA sequencing was performed using the PacBio Sequel IIe platform. High-quality reads were assembled by Canu v2.2 (7), and the assembled genomes were further refined by Circlator v1.5.5 (8) to identify circular contigs and to remove redundant non-circular contigs. This resulted in circular genomes (Table 1) with 31- to 32-fold coverages. A completeness check was performed by CheckM v1.0.18 (9), and the N50 quality was determined by Assembly stats ver1.01 (https://github.com/sanger-pathogens/assembly-stats). Genome ORF calling and annotation were performed by the NCBI Pipeline PGAP 6.9 (10). Default parameters were used for all tools.

**TABLE 1:** *M. lacunae* Ki8-1 and *M. stamsii* Pt1 genome information

	*M. lacunae* Ki8-1	*M. stamsii* Pt1
NCBI taxonomy ID	668570	1277351
DSM collection	DSM 22751	DSM 26304
Topology	circular	circular
Size (bp)	3,777,932	3,867,517
GC%	43.16	42.27
Protein-coding genes	3,402	3,632
16S number	4	4
tRNA number	57	53
Coverage	31.95×	31.12×
Raw reads	533,773	664,902
Average sheared size	8.9 Kb	9.5 Kb
Average read length	5,046.77 bp	6,192.91 bp
High-quality reads	14,330	12,373
Completeness	99.54%	99.02%
Contamination	1.31%	1.31%
N50	3,777,932 bp	3,867,517 bp
16S identity	99.76%^*a*^	99.72%^*b*^

^*a*^ Genomic 16S similarity to the published 16S NCBI accession no. AB517986  of *M. lacunae *Ki8-1.

^*b*^ Genomic 16S similarity to the published 16S NCBI accession no. HF569045 of *M. stamsii *Pt1.

The properties of the *M. lacunae* Ki8-1 and *M. stamsii* Pt1 genomes are shown in Table 1. Genomic 16S gene sequences for each strain were compared to the published 16S sequence of the described type strain using NCBI BLAST+ 2.17.0 (11) with close agreement.

Both the genomes are among the largest of all archaeal species sequenced, well above the 1.6 Mb average (12). Each genome exhibits a complete set of pathway genes for hydrogenotrophic methanogenesis but not for one-carbon or acetate. Each genome encodes multiple F420-dependent hydrogenase and formate dehydrogenase enzymes along with multiple cell envelope paralogs for the tubular sheath, surface layer, and flagellar proteins (6, 13, 14).

## Data Availability

The complete genome sequences of the strains have been deposited under the GenBank accession numbers GCA_046244385.1 for *M. stamsii* Pt1 and GCA_046195335.1 for *M. lacunae* Ki8-1. The raw sequencing reads have been deposited under the NCBI BioProject numbers SRR31625239 for *M. stamsii* Pt1 and SRR31625238 for *M. lacunae* Ki8-1. The Joint Genome Institute Integrated Microbial Genomics database (IMG) submission IDs for Pt1 and Ki8-1 are 338235 and 338236, respectively.
